# Managing human bites

**DOI:** 10.4103/0974-2700.55331

**Published:** 2009

**Authors:** Pradnya D Patil, Tanmay S Panchabhai, Sagar C Galwankar

**Affiliations:** Department of Medicine, Seth GS Medical College and KEM Hospital, Mumbai, India; 1 Department of Medicine, University of Louisville School of Medicine, Louisville, KY, USA; 2 Department of Global Health, University of South Florida, Tampa, FL, USA

**Keywords:** Antibiotic prophylaxis, human bites, human saliva, management

## Abstract

Human bites are frequently overlooked in making a diagnosis in the emergency room. They are particularly notorious due to the polymicrobial nature of human saliva inoculated in the wound and the risk they pose for transmission of infectious diseases. Early treatment, appropriate prophylaxis and surgical evaluation are the key to achieving desired treatment outcomes. Through this article, we have tried to summarize the diagnostic features, complications as well as the recommended treatment alternatives for human bites based on the current available evidence.

## INTRODUCTION

Mammalian bites account for almost 1% of emergency room (ER) visits annually in the United States,[1] the annual medical costs of managing these injuries being over $100 million.[2] Of these, human bites are particularly notorious for their propensity in causing infections at the site of the bite injury as well as posing a potential risk for transmission of systemic diseases. Hence, there is a need to pay special attention to the diagnosis and management of human bites, some of which can lead to disabling consequences.

## EPIDEMIOLOGY

Studies have found that human bites are more common among males, with the peak incidence being between 18 and 78 years of age (median - 28 years). In a study of 388 patients with human bites, more than half of the patients (50.3%) presented with bites on the hands or fingers, 23.5% on an extremity and 17.8% on the head or neck. Closed fist injuries accounted for only 7.7% of the bites, with occlusion bites accounting for the rest.[3] Majority of the patients (76.2%) presented to the ER within 12 h of injury. While most of the patients (77.3%) received antibiotics, 11.1% were admitted to the hospital. Patients who had greater odds of presenting more than 24 h after the bite were black (odds ratio [OR] 1.79), Hispanic (OR 2.68) and those who had a non-occupational bite (OR 3.87).[3] Patients with bite injuries are often intoxicated, making the process of obtaining a reliable history and conducting a thorough examination difficult. They are also frequently reluctant to admit to the cause of the injury, providing misleading histories. The rate of infection secondary to human bites is estimated to be about 10%.[4]

## PATHOPHYSIOLOGY

Human bites can be classified depending on the mechanism of injury into occlusion bites and the closed fist bite (or fight bite). Occlusion bites occur when the teeth are sunk into the skin with sufficient force to breach the integrity of the skin. Clenched fist injuries occur when a closed fist impacts another individual's teeth, leaving an injury over the dorsal aspect of the third, fourth or fifth metacarpophalangeal (MCP) joints, most classically over the third MCP. These are known to be among the worst human bites because although on initial examination they usually reveal a small, seemingly innocuous puncture that may be easily missed on examination, the penetration often injures the soft tissue, extensor tendon and sheath and may disrupt the MCP joint. Clenched fist bite wounds have been estimated to penetrate the MCP joint in as many as 52-62% bites, result in tendon injuries in 20%, cartilage injuries in 6% and bone injuries in 17-58%.[5] The reason these injuries are so prone to infection is that the extensor tendon and the MCP joint are relatively avascular structures and thus have a very limited ability to fight infection. After contact is made, the patient is likely to extend the hand, which facilitates the entry of oral bacteria along the extensor tendon deeper in the wound, which may then go on to invade the joint space or the tendon sheath extending to the wrist and dorsum of the hand.[4]

## COMPLICATIONS

Human saliva is known to contain as many as 50 species of bacteria with almost 10^8^ microbes/ml.[4] This is one of the reasons why human bites are believed to have higher rates of infection than other injuries. Other factors associated with higher rates of infection are delayed presentation to the ER, bites on the upper limbs and hand and bites on relatively avascular structures like the ear cartilage.[6] Bite wounds inflicted by children are known to have lower rates of infection owing to less diseased teeth or a lower incidence of gingivitis in children as compared with adults.[7] Clenched fist injuries can lead to septic arthritis of the MCP joint, which is a rapidly destructive process. Because the options for surgical reconstruction of MCP joints are limited, only 10% of the patients with septic arthritis following a human bite will regain their previous level of functioning,[4] with amputation rates as high as 7-20% being reported.[5] Recently, Staiano and Graham described two cases of fist bite injuries with retained fragments of tooth in the wound, leading to infection. In a multicenter study of infected human bites where 50 patients were studied and four cultures, including one anaerobic culture, were obtained from each patient, it was discovered that aerobic species alone were isolated in 44% of the wounds, anaerobes alone were isolated in 2% and both aerobes and anaerobes were isolated in 54% of the wounds.[8] The most common aerobic isolates were Streptococcus, Staphylococcus and Eikenella species. *Streptococcus anginosus* was the most common pathogen isolated and was found to contaminate 52% of human bites. The most common anaerobic pathogens were Prevotella and Fusobacterium species. Isolated cases of transmission of viral hepatitis,[9,10] herpes virus, tetanus,[11,12] Actinomyces and *Treponema pallidum*[13] through human bites have been reported in the literature. Although the consensus is that transmission of human immunodeficiency virus (HIV) via a human bite is unlikely, some anecdotal reports do exist.[14] Exposure to saliva alone is not considered a risk factor for viral transmission, although HIV may be present in the saliva (infrequently and at low levels). Salivary inhibitors render the virus non-infective in a majority of the cases.[15] Therefore, transmission of HIV is a risk when there is blood in the mouth of the person who bites and there is a breach in the skin of the victim.[16] There have also been reports of necrotizing fasciitis occurring after human bites. [17,18] Penile bites have been reported to transmit many serious infections such as Streptococcal toxic shock syndrome[19] and Fournier's gangrene.[20]

## DIAGNOSIS

A thorough examination of the bite wound in adequate lighting must be performed. The wound may be irrigated to facilitate the examination. The extent of damage to the soft tissue, depth of the bite, involvement of tendons, presence of infection or foreign bodies such as fragments of teeth must be assessed. Special care must be taken during the examination of fight bites and examination must be performed in the closed fist position so as to passively flex the fingers, making it easier to assess the damage to the extensor tendons. Due to their innocuous presentation, the safest course is to consider all injuries dorsal to the MCP joint to be fight bite wounds until proven otherwise.[4] History of time of injury, mechanism of injury, allergy status, time of last tetanus immunization and any known medical illnesses in the person who bit the patient must be obtained. All bite marks in a young child should raise suspicion of abuse. The normal distance between the maxillary canine teeth in adults is 2.5-4.0 cm. Any human bite marks with an intercanine distance greater than 3.0cm were likely inflicted by an adult. Suspicious wounds should be photographed next to a ruler, a thorough skin examination should be carried out and appropriate consultations should be made.[21] Radiographs must be obtained in all clenched fist injuries and penetrating scalp wounds in children to rule out fractures, presence of foreign bodies or teeth fragments in the wound or, in late cases, osteomyelitis. As demonstrated by Staiano and Graham, retained fragments of teeth in the wound can prove to be very dangerous; hence, every attempt must be made to rule out the presence of foreign bodies in the bite wound.[22]

## MANAGEMENT

### Initial management

Most patients attempt some form of self-treatment before seeking medical attention.[23] The first step in management is cleansing of the wound and irrigation with saline, 1% povidone iodine or tap water at body temperature in a 20 ml or larger syringe and a 19-gauge needle, which will provide a high-pressure jet that serves to reduce the bacterial inoculum and debride the wound. However, irrigation must be in the direction of the puncture wound and care must be taken not to inject into the tissue or inflict further damage.[7] This should be followed by debridement of the devitalized tissue if required. Debridement of necrotic or devitalized tissue should be done cautiously, with the realization that residual defects or problems with wound closure may occur. As a rule, puncture wounds should not be debrided.[24] Even the simplest of wounds require copious irrigation and wound toilet. If the bite wound is on a limb, it is elevated. If the bite wound involves a joint, the joint must be washed out and immobilized.[25] It is recommended that tetanus immunoglobulin and tetanus toxoid should be administered to patients with a history of two or fewer immunizations.[26] Cultures (including culture for anaerobic organisms) should be obtained from deep within the wound if it is clinically infected. Early wound cultures are rarely helpful. Blood cultures may be obtained if there is systemic toxicity or the patient is immunocompromised.[26]

### Role of inpatient therapy

Table 1 lists patients who are at a high risk of infection, who must be considered for inpatient therapy.[24] In addition, patients with systemic manifestations of infection, such as fever, chills, elevated white blood cell count, those who fail to improve on initial outpatient therapy, those with a high likelihood of non-compliance (mentally handicapped, homeless people or alcoholics) and those with infected hand wounds, must be admitted. Any patient with an injury severe enough to require operative exploration should be observed at least overnight postoperatively.

**Table 1:** Patients with bite wounds who may benefit from inpatient therapy

Hand, foot or face wounds	Patients > 50 years
Scalp involvement	Immunosuppressed patients
Bone/joint involvement	Chronic alcoholism
Puncture wounds	Diabetes mellitus
Crush injuries	Vascular disease
Delayed treatment	Pre-existing edema of the affected extremity

### Antibiotic prophylaxis

There are no clear guidelines for the initiation of antibiotic prophylaxis after a human bite. There are only two randomized clinical trials to date to study the advantage of prophylactic antibiotics in human bite injuries. In the earlier trial,[27] patients with bites on the hand (bites involving joints or tendons were excluded) were randomized to either a placebo, an oral cephalosporin or a combination of parenteral cephalosporin and penicillin. Forty-seven percent of the patients on the placebo developed infection whereas not a single patient on prophylactic antibiotics developed infection. A more recent trial,[2] which included only patients whose bite injuries did not penetrate deeper than the epidermis, randomized to a placebo or an oral cephalosporin plus penicillin showed no statistical difference in the infection rates among both groups. Thus, it is probably appropriate to initiate prophylactic antibiotics in patients whose bite wounds are contaminated, those with puncture wounds, bites on the hands or those involving structures such as joint capsules, tendons and cartilage.[21] Antibiotics should cover common pathogens such as Streptococcus (especially *Streptococcus anginosus*), Staphylococcus and Eikenella and provide broad anaerobic cover. According to Talan *et al.*, 50% of the Provetella isolated and 80% of the Staphylococci isolated from human bite wounds were β-lactamase producers. Eikenella isolates were relatively resistant to clindamycin, erythromycin, aminoglycosides and anti-Staphylococcal penicillins and first-generation cephalosporins.[8] *In vitro* studies demonstrated that amoxicillin clavulanate and moxifloxicin demonstrated excellent activity against these isolates. If patients are to receive antimicrobial prophylaxis, the first dose ideally may be given parenterally to obtain effective tissue levels as quickly as possible, although there isn't sufficient evidence to support or refute that this is a better practice than oral antibiotics alone. Appropriate oral antibiotics should be given and continued for 3-5 days.[28] The antibiotics indicated for the prophylaxis and treatment of infected human bites are mentioned in Table 2. When cellulitis is already present, a therapeutic course of 10-14 days may be necessary, extended to 3 weeks for tenosynovitis, 4 weeks for septic arthritis and 6 weeks for osteomyelitis. In practice, intravenous therapy is continued until the C-reactive protein falls to less than 50 mg/L, after which oral antibiotics may be given. If the C-reactive protein does not fall rapidly or remains static, clinical re-appraisal and a second debridement is advisable, particularly with joint space infections.[26] We also conducted a literature search for evidence with regard to the management of self-mutilation injuries. However, other than anecdotal case reports, other data were not retrieved. We however believe that the management of self-mutilation injuries in psychiatric patients should be on similar lines to the management of other human bites.

**Table 2:** Antibiotic management of human bites[1]

Antibiotic therapy	
Hand bites, high risk bites	
Adults	First parental dose, ampicillin–sulbactam (3 g) or cefoxitin (1 g) or ertapenam (1 g) followed by amoxicillin–clavulanate (875/125 mg PO q 12 h) for 3-5 days
	or
	First parental dose of clindamycin (600 mg) followed by clindamycin (300 mg PO q 8 h) plus a fluoroquinolone (ciprofloxacin, 500 mg PO q 12 h, or levofloxacin, 750 mg/d PO, or moxifloxacin, 400 mg/d PO) for 3-5 days,
	or
	If parental therapy cannot be given, appropriate oral antibiotics (e.g., amoxicillin–clavulanate, 875/125 mg PO q 12h) for 3-5 days
Children	First parental dose: Ampicillin–sulbactam (50 mg/kg as ampicillin to a maximum of 3 g) followed by amoxicillin–clavulanate (>40 kg: 875/125 mg PO q 12 h; >3 mo and <40 kg: 45 mg/kg/d PO divided q 12 h or 40 mg/kg/d PO divided q 8 h) for 3-5 days
	or
	First parental dose: Clindamycin (5-10 mg/kg IV to a maximum of 600 mg) followed by clindamycin (10-30 mg/kg/d PO divided q 6-8 h to a maximum of 300 mg/dose) plus trimethoprim–sulfamethoxazole (8-10 mg/kg of trimethoprim/d PO divided q 12 h) for 3-5 days
Human bites, infected	
Adults	Ampicillin–sulbactam (3 g IV q 4-6 h) or cefoxitin (1 g IV q 6-8 h) or ertapenam (1 g/d IV)
	Subsequent oral therapy or initial therapy for minor infection: Amoxicillin–clavulanate (875/125 mg PO q 12 h)
Children	Ampicillin–sulbactam (100-200 mg/kg/d IV divided q 6 h to a maximum of 3 g/dose)
	or
	Cefoxitin or ticarcillin–clavulanate can be given alternatively
	Subsequent oral therapy or initial therapy for minor infection: Amoxicillin–clavulanate (> 40 kg: 875/125 mg PO q 12 h; > 3 mo and < 40 kg: 45 mg/kg/d divided q12 h or 40 mg/kg/d divided q 8 h)

### Post exposure prophylaxis for HIV and hepatitis B

HIV PEP is not routinely indicated after a human bite. PEP is to be started only in rare circumstances where there has been an exposure to a known HIV-infected source with a high viral load and the exposure involves significant blood transfer, a deep wound, entry into a blood vessel, etc.[29] It may be prudent to obtain a baseline HIV serological test with a 6-month follow-up test.[7] If possible, the assailant should be tested for HB surface antigen and HB envelope antigen. If positive, the patient should be given a single dose of HB immunoglobulin and an accelerated course of HB vaccine (doses at 0, 1 and 2 months), unless the patient is known to be immune.[25] However, the rate of transmission of HB virus is not high enough to warrant routine prophylaxis in bites from an unknown source.

### Role of surgical management

Surgical management of human bites ranges from simple surgical exploration of the wound to repair of complex structures under the microscope. Children and people who are mentally handicapped may require exploration under anesthesia to facilitate proper examination. Indications for surgical intervention are the presence of severe soft tissue infection, abscess, joint penetration, underlying fracture, tendon rupture, osteomyelitis, tenosynovitis, septic arthritis, neurovascular compromise and presence of a foreign body such as tooth fragments in the wound. The decision to surgically close a human bite wound depends on many factors. Usually, human bites are contaminated wounds and are thus closed by delayed primary or secondary suturing. [30] A prospective cohort study by Chen *et al.* demonstrated that primary closure of bite wounds is associated with higher rates of infection (6%) as compared with other sutured wounds in the same institution (3.4%).[31] Although primary repair of bite wounds is associated with higher rates of infection, it is still indicated for bite injuries where cosmetic outcome is important. Because bites on the face are associated with more bleeding, they are at a lower risk of infection following primary closure. Thus, primary closure of all uninfected wounds of the face is indicated, whereas debridement and delayed closure may be performed in certain high-risk or already infected wounds.[32] Donkor and Bankas studied 30 patients who presented with human bites of the face and noted that a thorough debridement followed by primary closure, direct suturing, a local flap or skin grafting on the day of presentation resulted in 90% complete wound healing.[33] A recent case series indicated that locally injected hyaluronidase may aid in decreasing the edema and increasing perfusion in face bites that present late thus permitting wound closure without tension[34] Bite wounds must be closed using a standard percutaneous closure technique with a non-absorbable suture, such as a monofilament nylon or polypropylene. Multiple layer closures and subcutaneous sutures are to be avoided unless absolutely necessary. Surgical closure of non-facial wounds, especially deep punctures, wounds more than 24 h old, bites to the hand and clinically infected wounds is not indicated due to an increased rate of infection. Adhesive strips and a delayed surgical closure may be performed in such cases.[24] In all cases where closure of the wound is performed, the patient should be called for follow-up after 48-72 h. Because hand injuries are associated with a higher rate of infection, the consult of a hand surgeon must be sought. A surgeon should be consulted in all cases of fight bite injuries or those involving injuries to the tendons or deeper structures or when complications such as infection develop. If hand wounds are infected, physical therapy is typically initiated 3 to 5 days after the infection resolves to regain function of the affected hand.[25] Inpatient therapy is advised for patients with associated fractures, septic arthritis or involvement of the joint capsule, tendons, etc. Proper instructions on discharge and assuring follow-up are as important as the initial care. The patient must be educated about wound care and signs of infection and asked to follow-up immediately in the eventuality that an infection develops.

## CONCLUSION

Human bites are potentially dangerous wounds and constitute a significant cause of morbidity. Emergency physicians should be well acquainted with the evaluation and proper management of human bites to avoid complications. Early treatment, appropriate prophylaxis and surgical evaluation are key to achieving desired treatment outcomes. Due to the polymicrobial nature of these wounds and prevalence of antibiotic resistance, emergency physicians should be aware of the common organisms involved and their susceptibility to commonly used antibiotics.
